# Tape Worm Diagnosis by Urinalysis

**Published:** 1896-09

**Authors:** W. W. Pugh

**Affiliations:** Hearne, Texas


For the Texas Medical Journal.

### TAPE-WORM—DIAGNOSES BY URINALYSIS.

BY W. W. PUGH, M. D., HEARNE, TEXAS.

IN MAKING an examination of urine for a man desiring to become a member of a lodge for which I was examining physician, I found his specific gravity 1005, no albumen, no sugar. I was a little surprised in not finding albumen, as we generally do in low specific gravity. In six or seven days the party came to me and stated he wanted me to remove a tape-worm from him, as he was passing joints of it. The idea struck me; probably this accounts for the low specific gravity. So I removed the worm, and in five days re-examined his urine and found specific gravity 1020, no albumen, no sugar. I merely mention this case and would like for other doctors to make urinalysis in tape-worm case, and see if this is a sure diagnostic point in tape-worm subjects. If it is, then I have made a discovery.

